# Asymmetric reverse buffering patterns of source-specific social support in the associations between depression, anxiety, and suicidal ideation among college students

**DOI:** 10.3389/fpsyt.2026.1930307

**Published:** 2026-09-30

**Authors:** Xiaoxia Tian, Yanli Shi, Pengcheng Wei, Canxue Zhao, Ruimin Cheng, Yalei Zhou

**Affiliations:** 1 School of Medicine and Health, Wuhan Polytechnic University, Wuhan, China; 2 Mental Health Education and Counseling Center, Wuhan Polytechnic University, Wuhan, China

**Keywords:** anxiety, asymmetric buffering, college students, depression, reverse buffering effect, social support, suicidal ideation

## Abstract

**Objective:**

Suicidal ideation remains a major mental health concern among college students. This study examined whether perceived social support from family, friends, and significant others differentially moderates the associations of depressive and anxiety symptoms with suicidal ideation, with particular attention to a potential asymmetric buffering pattern.

**Methods:**

A cross-sectional survey was conducted among 504 college students using the Patient Health Questionnaire-9 (PHQ-9), Generalized Anxiety Disorder-7 (GAD-7), Multidimensional Scale of Perceived Social Support (MSPSS), and Self-Rating Idea of Suicide Scale (SIOSS). Primary analyses used full-sample continuous interaction models in which depressive and anxiety symptoms were entered simultaneously. Robustness was assessed using the PHQ-8, while sensitivity analyses examined depressive and anxiety symptoms separately and further explored the associations across social support subgroups.

**Results:**

After adjustment for demographic covariates, perceived social support from family, friends, and significant others showed significant buffering effects on the positive association between depressive symptoms and suicidal ideation. In contrast, these sources of social support showed significant positive interaction effects with anxiety symptoms, indicating a reverse-buffering pattern in which higher perceived social support was associated with a stronger positive association between anxiety symptoms and suicidal ideation. The PHQ-8 analysis showed generally consistent interaction patterns. However, the single-predictor and subgroup sensitivity analyses showed attenuation or variability in some interaction effects.

**Conclusions:**

The findings suggest a potentially asymmetric pattern in the moderating role of perceived social support, with conventional buffering observed for depressive symptoms but reverse buffering observed for anxiety symptoms. However, the observed interaction pattern was sensitive to model specification, and the findings should therefore be considered preliminary and require replication in future studies. These results highlight the importance of considering both the source of social support and the specific emotional pathway when examining the relationship between social support and suicidal ideation among college students.

## 1 Introduction

### 1.1 Background and practical significance

Suicidal ideation is a well-established precursor of suicidal behavior and a major contributor to premature mortality among individuals aged 15–29 (1). College students represent a particularly vulnerable population due to the extensive developmental transitions they experience (2), including psychological maturation, identity formation, and social role adjustment (3). Within this context, multiple stressors such as academic demands, interpersonal difficulties, educational pressures, and employment-related concerns contribute to elevated levels of depressive and anxiety symptoms (4). As a result, suicidal ideation is more prevalent among college students than in the general adult population, highlighting suicide prevention as a critical priority for university-based mental health services (5).

Current psychological interventions in university settings are largely grounded in the traditional assumption that higher levels of social support are uniformly protective against emotional distress and suicidal risk (6). Accordingly, intervention strategies often focus on increasing overall perceived social support, with limited consideration of potential functional differences across distinct support sources. In addition, relatively little attention has been given to potential divergences in the mechanisms linking depression and anxiety to suicidal ideation. This has resulted in intervention frameworks that may overlook pathway-specific processes and source-specific effects, thereby limiting the precision of suicide prevention strategies in student populations.

Social support has long been conceptualized as a core psychosocial resource and a protective factor that alleviates negative affect and reduces suicide risk (7). However, accumulating evidence has indicated that its effects are not uniformly beneficial, with a growing body of research reporting paradoxical findings (8). In particular, under certain conditions—such as when support is excessive, mismatched, or incongruent with individuals’ psychological needs—higher levels of perceived support may be associated with adverse psychological outcomes (9). These mixed findings suggest that the protective function of social support is not invariant. Instead, its effects may vary as a function of support source, level of support, and the specific emotional pathways through which psychological distress translates into suicidal ideation.

From the perspective of source-specific social support heterogeneity, this study investigates the asymmetric buffering mechanisms of distinct sources of social support in the associations among depression, anxiety, and suicidal ideation in college students. The findings contribute to a more nuanced understanding of social support processes and offer empirical evidence for the development of targeted, precision-based suicide prevention strategies in university settings.

### 1.2 Literature review and research gap

#### 1.2.1 The classical stress-buffering model of social support and its limitations

According to the classical stress-buffering model proposed by Cohen, social support can mitigate the adverse effects of stress on psychological well-being. Within this framework, social support is conceptualized as a protective resource that attenuates the impact of stressful experiences on mental health outcomes, typically operating in a uniform and symmetric buffering manner.

This assumption is supported by a substantial body of empirical research demonstrating that social support is negatively associated with depression, anxiety, and suicidal ideation (7). Higher levels of perceived support have been consistently associated with lower levels of emotional distress and reduced suicide risk. Accordingly, the buffering hypothesis has been widely adopted as a central theoretical framework for understanding the protective role of social relationships in mental health (6).

#### 1.2.2 Research progress on the heterogeneity of social support sources

Social support is typically conceptualized as comprising three source-based categories: family support, friend support, and significant-other support (10). These sources differ in terms of stability, emotional closeness, and contextual adaptability. Family support is generally characterized by long-term stability and a foundational function of emotional security. Friend support primarily reflects peer-based empathy and responsiveness to daily stressors. In contrast, significant-other support represents a more flexible and context-dependent form of emotional and instrumental assistance (11).

Existing research has primarily examined the differential effects of social support sources on general mental health outcomes. However, less attention has been given to whether distinct sources of support operate through different mechanisms across specific emotional pathways, particularly those associated with depression and anxiety. As a result, the potential heterogeneity in source-specific functional roles across emotional processes remains underexplored, limiting a more mechanistic understanding of how social support influences suicidal ideation.

#### 1.2.3 Research gaps in reverse buffering effects and asymmetric mechanisms

The emergence of the social support paradox and support mismatch perspectives has accumulated evidence suggesting that social support is not uniformly beneficial (8, 9). In certain contexts, excessive or poorly matched support may produce adverse effects, a phenomenon conceptualized as reverse buffering. This pattern indicates that social support may, under specific conditions, strengthen rather than attenuate the association between psychological distress and maladaptive outcomes.

Notwithstanding these advances, several important gaps remain in the extant literature. First, few studies have systematically examined whether reverse buffering effects vary as a function of social support source, including family, friends, and significant others, limiting understanding of source-specific triggering patterns. Second, prior research has seldom differentiated between depression- and anxiety-related cognitive and affective pathways (12), resulting in limited evidence regarding potential asymmetry across these emotional dimensions. Third, the boundary conditions under which social support shifts from a buffering to an exacerbating role remain insufficiently specified (9), thereby constraining the explanatory power of the classical symmetric buffering model in accounting for empirical inconsistencies.

Collectively, these gaps underscore the need to further investigate source-specific and pathway-dependent asymmetric mechanisms of social support in relation to suicidal ideation among college students.

#### 1.2.4 Definition of core concepts

The heterogeneity of social support sources refers to systematic differences among family, friend, and significant-other support in their underlying psychological mechanisms (10), contextual applicability, risk activation conditions, and protective functions. Rather than being functionally equivalent, different sources of social support may exert distinct moderating effects on the association between negative emotional states and suicidal ideation, with effects varying across support sources.

The term asymmetric reverse buffering effect refers to a differentiated moderating pattern in which the associations between emotional distress (i.e., depression and anxiety) and suicidal ideation vary as a function of both the type and level of social support. In the depression pathway, social support typically exhibits a conventional buffering effect that attenuates the association with suicidal ideation. In contrast, within the anxiety pathway, higher levels of social support may, under specific conditions, strengthen the association between anxiety and suicidal ideation, reflecting a reverse buffering pattern. Moreover, this asymmetric pattern may vary across different sources of social support, suggesting source-dependent differences in both the direction and magnitude of buffering effects.

### 1.3 Research aims and research questions

Grounded in the heterogeneity of social support sources, the present study extends the classic buffering model by moving beyond its conventional assumption of symmetric protective effects. We systematically examined the moderating roles of three sources of social support in the associations of depression and anxiety with suicidal ideation among college students, with particular attention to the potential for asymmetric reverse buffering effects and source-specific heterogeneity. Multiple sensitivity analyses were also conducted to assess the robustness and potential boundaries of these effects.

Accordingly, the present study addressed three core research questions. First, does social support differ by source in its moderating role, and do different sources of social support show distinct patterns of moderation in the associations between emotional distress and suicidal ideation? Second, do the depression–suicidal ideation and anxiety–suicidal ideation associations exhibit asymmetric reverse buffering effects of social support? Third, do subgroup sensitivity analyses provide preliminary evidence regarding the potential conditions and patterns associated with these asymmetric effects across different levels of social support?

### 1.4 Research hypotheses

To examine source-specific heterogeneity and asymmetry in the moderating role of perceived social support, the following hypotheses were proposed:

H1: Depressive and anxiety symptoms are positively associated with suicidal ideation among college students.

H2: The moderating effects of perceived social support may vary across support sources (family, friends, and significant others) in the associations of depressive and anxiety symptoms with suicidal ideation.

H3: Perceived social support shows an asymmetric moderating pattern, with interaction effects consistent with conventional buffering in the depression–suicidal ideation association and reverse buffering in the anxiety–suicidal ideation association.

Additional subgroup analyses based on different levels of perceived social support were conducted as exploratory sensitivity analyses rather than confirmatory hypothesis tests. These analyses were intended to explore the potential boundaries and variability of the observed moderation patterns.

### 1.5 Theoretical framework

**Figure 1** presents the theoretical framework of the present study, which builds on Cohen’s stress-buffering model and its asymmetric extension (6). Within this framework, depression and anxiety are conceptualized as two representative forms of internalizing psychological distress that serve as the primary predictors of suicidal ideation among college students. Family support, friend support, and significant-other support are specified as moderating variables to examine whether different sources of social support differentially influence the associations between depression and suicidal ideation, as well as between anxiety and suicidal ideation.

**Figure 1:**
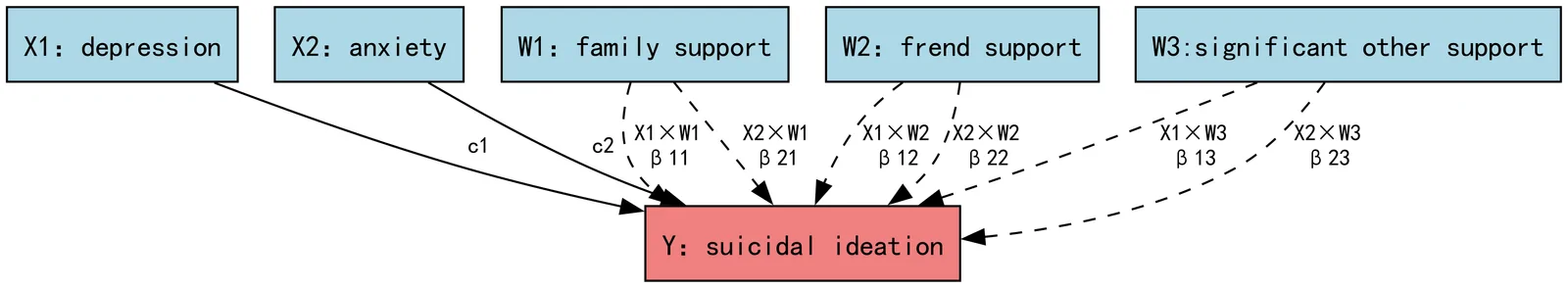
Theoretical model of source-specific social support in the associations between depression, anxiety, and suicidal ideation. Depression (X1) and anxiety (X2) were specified as the primary predictors of suicidal ideation (Y). Family support (W1), friend support (W2), and significant-other support (W3) were hypothesized to moderate the relationships between emotional distress and suicidal ideation. Solid lines denote direct effects, whereas dashed lines denote moderating effects.

In contrast to the classical stress-buffering model, which assumes that social support exerts a uniform and symmetric protective effect, the proposed framework posits that the buffering function of social support is both source-specific and pathway-dependent. Accordingly, the direction and magnitude of the moderating effects are expected to vary across support sources and emotional pathways, giving rise to asymmetric buffering patterns.

The proposed framework provides a conceptual basis for explaining how different sources of social support differentially influence the development of suicidal ideation among college students. It also offers an integrated framework for examining the heterogeneity of social support sources and testing asymmetric reverse buffering effects across the depression–suicidal ideation and anxiety–suicidal ideation pathways.

## 2 Methods

### 2.1 Study design and participants

This study employed a cross-sectional questionnaire survey. From March to May 2025, an online survey was conducted among undergraduate and postgraduate students from multiple general higher education institutions in China. The questionnaire was administered through Wenjuanxing, a widely used online survey platform in China, using convenience sampling. Recruitment was primarily conducted through university psychological associations, class-based WeChat groups, and student social networking channels, with participants recruited from multiple provinces across China.

A total of 614 questionnaires were initially collected. During data cleaning, questionnaire quality was assessed using predefined quality-control criteria. Questionnaires were excluded for the following reasons: (1) completion time of less than 3 minutes (n = 68); (2) missing data exceeding 20% of the core variables (n = 12); (3) highly repetitive or patterned responding (n = 10); (4) failure on an attention-check item (n = 5); or (5) failure to meet the validity criterion of the SIOSS concealment subscale (n = 15). These five exclusion categories accounted for all 110 excluded questionnaires. After screening, 504 questionnaires were retained for analysis, representing a proportion of usable questionnaires of 82.1% (504/614). No monetary or material incentives were provided for participation.

The final sample comprised 504 valid respondents. Of these, 360 (71.4%) were female and 144 (28.6%) were male. More than half of the participants were aged 19–22 years (56.6%). Regarding place of origin, 269 participants (53.4%) were from urban areas and 235(46.6%) were from rural areas. Participants were distributed across different academic years, with fourth-year undergraduates accounting for the largest proportion (36.5%), followed by postgraduate students (18.5%). In terms of academic discipline, 57.9% of participants majored in humanities and social sciences, while 35.9% were enrolled in science-related disciplines.

Based on the established cutoff scores of the Self-Rating Idea of Suicide Scale (SIOSS), participants were classified into two groups: a suicidal ideation group (SIOSS ≥ 12, n = 233) and a non-suicidal ideation group (SIOSS < 12, n = 271) (13). Group comparisons indicated a significant difference in sex between the two groups (χ² = 5.108, p = .024), whereas no significant differences were observed in academic year, only-child status, or monthly living expenses (all p >.05).

### 2.2 Inclusion and exclusion criteria

The inclusion criteria were as follows: (a) being an enrolled undergraduate, master’s, or doctoral student at a general higher education institution in China; (b) having sufficient comprehension to independently and accurately complete the questionnaire and self-report measures; and (c) having read the online informed consent form before participation, understood the relevant study procedures and requirements, and voluntarily agreed to participate. Students younger than 18 years were not included in the present study.

Participants were excluded from the study if they: (a) failed the embedded attention-check item; (b) provided missing, incomplete, or logically inconsistent responses on key study variables; (c) showed invalid response patterns, such as invariant responding (e.g., selecting the same response option throughout the questionnaire), excessively short completion times, or other aberrant response behaviors; or (d) self-reported a diagnosis of a severe psychiatric disorder or an organic neurological disorder that could substantially compromise the validity of questionnaire responses.

### 2.3 Measures

#### 2.3.1 Demographic characteristics

Participants provided self-reported demographic information, including sex, age, place of origin (urban or rural), only-child status, monthly living expenses, academic year, and academic discipline.

#### 2.3.2 Patient health questionnaire-9

Depressive symptoms experienced during the preceding two weeks were assessed using the Patient Health Questionnaire-9 (PHQ-9) (14). The PHQ-9 consists of nine items rated on a 4-point Likert scale ranging from 0 (not at all) to 3 (nearly every day), with higher scores indicating more severe depressive symptoms. The PHQ-9 has demonstrated good reliability and validity in Chinese college student populations and is widely used to assess depressive symptoms in young adults (15). In the present study, the scale showed excellent internal consistency (Cronbach’s α = .92).

#### 2.3.3 Generalized anxiety disorder-7

Anxiety symptoms over the preceding two weeks were assessed using the Generalized Anxiety Disorder-7 (GAD-7) (16). The GAD-7 is a brief self-report instrument comprising seven items, each rated on a 4-point Likert scale ranging from 0 (not at all) to 3 (nearly every day), with higher total scores indicating greater severity of anxiety symptoms. The scale has demonstrated excellent psychometric properties, including strong reliability and validity, and has been widely employed to assess anxiety symptoms among Chinese college students (17). In the present study, the GAD-7 exhibited excellent internal consistency (Cronbach’s α = .93).

#### 2.3.4 Multidimensional scale of perceived social support

The Multidimensional Scale of Perceived Social Support (MSPSS) was used to assess perceived social support (10). The MSPSS is a 12-item self-report instrument comprising three subscales: Family Support, Friend Support, and Significant-Other Support, with each subscale consisting of four items. Items are rated on a 7-point Likert scale, with higher scores indicating greater levels of perceived social support. The MSPSS enables the assessment of support from multiple social sources and provides a robust framework for examining the heterogeneity of source-specific social support. In the present study, the internal consistency coefficients (Cronbach’s α) were.80 for Family Support,.90 for Friend Support, and.90 for Significant-Other Support, indicating good to excellent reliability.

#### 2.3.5 Self-rating idea of suicide scale

The presence of suicidal ideation was assessed using the Self-Rating Idea of Suicide Scale (SIOSS) (13). The SIOSS is a 26-item self-report instrument comprising four dimensions: despair, optimism, sleep, and concealment. Each item is scored dichotomously, and the optimism items are reverse-coded according to the standardized scoring procedure. The concealment dimension is used as a validity indicator rather than being included in the suicidal ideation score. According to the standard scoring criterion, a concealment score of ≥4 indicates an unreliable response and warrants exclusion. Accordingly, 15 questionnaires with concealment scores ≥4 were excluded from the analysis. The suicidal ideation score was calculated as the sum of the despair, optimism, and sleep subscales. Higher total scores indicate greater severity of suicidal ideation, with scores of 12 or above indicating the presence of suicidal ideation. The SIOSS has been widely used in Chinese populations and has demonstrated satisfactory psychometric properties. In the present study, the scale exhibited acceptable internal consistency (Cronbach’s α = .75).

### 2.4 Procedure

The data were collected from March to May 2025 through an anonymous online questionnaire. All participants were aged ≥18 years and provided electronic informed consent before completing the questionnaire. Participation was voluntary, and participants were informed that they could withdraw at any time without penalty. Given that the questionnaire included an assessment of suicidal ideation, information on psychological crisis support and referral resources was provided to participants both at the beginning and after completion of the survey.

Because the data collection had been completed before the ethics review, a retrospective ethical review was conducted by the Biomedical Ethics Committee of Wuhan Polytechnic University, which approved the study on August 27, 2026 (approval No. BME-2026-1-33). The study complied with the Declaration of Helsinki (18).

### 2.5 Statistical analysis

The primary aim of the statistical analyses was to examine the moderating role of perceived social support in the associations of depressive and anxiety symptoms with suicidal ideation, with interaction effects examined separately for family, friend, and significant-other support. The analyses also described the observed interaction patterns across emotional symptom dimensions and support sources; however, no formal statistical comparisons between interaction coefficients across support sources were conducted. Robustness and sensitivity analyses were additionally performed to assess the stability and potential boundaries of the observed effects. All statistical analyses were performed using IBM SPSS Statistics 27.0. The specific analytical procedures were as follows.

#### 2.5.1 Preliminary analyses and correlation analysis 

Harman’s single-factor test was first conducted to assess potential common method bias (19). Descriptive statistics were calculated for depressive symptoms, anxiety symptoms, perceived social support, its three sources (family, friends, and significant others), and suicidal ideation. Pearson correlation analyses were then conducted to examine associations among the core variables and provide a preliminary basis for subsequent regression analyses.

Hierarchical regression, sensitivity, and robustness analyses were subsequently performed to examine the associations of depressive and anxiety symptoms with suicidal ideation and the moderating roles of the three sources of perceived social support.

#### 2.5.2 Hierarchical regression analysis

The full-sample continuous interaction model simultaneously including depressive and anxiety symptoms was used as the primary model for testing moderation. Mean-split and 27% extreme-group analyses were treated as secondary exploratory sensitivity analyses rather than confirmatory tests of the primary hypotheses.

All continuous variables were mean-centered before constructing interaction terms. VIFs were examined to assess multicollinearity. The VIFs for the main-effect predictors were all below 5, while the highest VIF for an interaction term was 6.4. When an interaction term was statistically significant, simple slope analyses were conducted at the mean and ±1 SD of the moderator, with corresponding 95% confidence intervals (CIs). Moderation was determined based on the interaction-term coefficient and its CI rather than differences in the significance of simple slopes across moderator levels.

Suicidal ideation was assessed using the Self-Rating Idea of Suicide Scale (SIOSS). The mean score was 11.58 (SD = 3.93; range = 0–21), with skewness of 0.28 and kurtosis of −0.86, indicating an approximately normal distribution. All tests were two-tailed, with p < 0.05 considered statistically significant. For statistically significant interaction terms, conditional effects were examined at the mean and ±1 SD of the continuous moderator, with B, SE, 95% CI, and p values reported.

#### 2.5.3 Robustness and sensitivity analyses

To address potential conceptual overlap between PHQ-9 item 9 and suicidal ideation, the PHQ-9 was replaced with the PHQ-8, and the primary moderation model was repeated in the full sample.

Given the high correlation between depressive and anxiety symptoms, two additional sensitivity models were examined separately: one including depressive symptoms, social support, and their interaction terms, and the other including anxiety symptoms, social support, and their interaction terms. These analyses assessed the extent to which the observed moderation patterns depended on the simultaneous inclusion of both emotional symptom dimensions.

#### 2.5.4 Subgroup sensitivity analyses

Subgroup regression analyses were conducted using a mean-split approach and an extreme-group approach based on the lower and upper 27% of the social support distribution. Because categorizing a continuous moderator reduces information and statistical power, particularly in the 27% extreme-group analysis, these analyses were considered exploratory and were used only to examine the potential boundaries and variability of the observed associations (20).

Perceived social support was assessed using the Multidimensional Scale of Perceived Social Support (MSPSS). Based on the sample mean (M = 45.83, SD = 12.65), participants were classified into low (total score <45.83, n = 187) and high (total score ≥45.83, n = 317) social support groups. These groups were used for secondary subgroup analyses.

Because multiple interaction terms involving different sources of social support were examined, the analyses were potentially subject to an increased Type I error rate. No adjustment for multiple comparisons was applied, and raw p values are reported. Therefore, subgroup and multiple-interaction findings should be interpreted cautiously and considered exploratory pending replication in independent samples.

#### 2.5.5 Ethical considerations

The study was conducted through an anonymous online survey, and data were collected between March and May 2025. Before completing the questionnaire, all participants were informed about the purpose and procedures of the study, the voluntary nature of participation, and their right to withdraw at any time. Electronic informed consent was obtained from all participants prior to participation. Because the questionnaire included questions assessing suicidal ideation, mental-health counseling hotline and crisis-support information was provided both at the beginning and after completion of the questionnaire.

The study protocol was retrospectively reviewed and approved by the Biomedical Ethics Committee of Wuhan Polytechnic University (Approval No. BME-2026-1-33) on August 27, 2026.

## 3 Results

### 3.1 Common method bias

The unrotated exploratory factor analysis showed that the first common factor accounted for 34.562% of the total variance, which was below the commonly used 40% threshold. However, Harman’s single-factor test is only a limited diagnostic approach and cannot completely rule out the possibility of common method bias (21).

### 3.2 Baseline characteristics of the sample

The final analyses included a total of 504 college students, comprising 271 participants (53.8%) in the non-suicidal ideation group and 233 participants (46.2%) in the suicidal ideation group. Chi-square analyses revealed a significant between-group difference in sex (χ² = 5.108, p = .024), with a lower proportion of females in the suicidal ideation group (66.5%) than in the non-suicidal ideation group (75.6%). No statistically significant between-group differences were observed for age, only-child status, place of origin, monthly living expenses, academic year, or academic discipline (all p >.05), indicating that the two groups were generally comparable with respect to baseline demographic characteristics (**Table 1**). 

**Table 1:** Demographic characteristics of participants by suicidal ideation status.

Variables	Category	Negative group (N = 271) n(%)	Positive group (N = 233) n(%)	χ²	df	*P*
Gender	Male	66 (24.4%)	78 (33.5%)	5.108	1	0.024*
	Female	205 (75.6%)	155 (66.5%)			
Age	18-20	64 (23.6%)	52 (22.3%)	0.168	3	0.983
	21–23	153 (56.5%)	132 (56.7%)			
	24–25	42 (15.5%)	38 (16.3%)			
	26 and over	12 (4.4%)	11 (4.7%)			
Whether an only child	Yes	92 (33.9%)	85 (36.5%)	0.353	1	0.553
	No	179 (66.1%)	148 (63.5%)			
Place of origin	Urban	144 (53.1%)	125 (53.6%)	0.013	1	0.909
	Rural	127 (46.9%)	108 (46.4%)			
Monthly living expenses	≤1,000 yuan	10 (3.7%)	19 (8.2%)	7.344	4	0.119
	1,001–2,000 yuan	163 (60.1%)	146 (62.7%)			
	2,001–3,000 yuan	74 (27.3%)	52 (22.3%)			
	3,001–4,000 yuan	21 (7.7%)	12 (5.2%)			
	≥4,001 yuan	3 (1.1%)	4 (1.7%)			
Grade	Freshman	19 (7.0%)	17 (7.3%)	2.078	4	0.721
	Sophomore	55 (20.3%)	39 (16.7%)			
	Junior	51 (18.8%)	46 (19.7%)			
	Senior	93 (34.3%)	91 (39.1%)			
	Graduate Student	53 (19.6%)	40 (17.2%)			
Major	Liberal Arts	154 (56.8%)	138 (59.2%)	1.408	3	0.704
	Science	98 (36.2%)	83 (35.6%)			
	Engineering	10 (3.7%)	8 (3.4%)			
Variables	Other	9 (3.3%)	4 (1.7%)

Data are presented as n (%). The negative group and positive group were classified according to the cutoff score of the Self-Rating Idea of Suicide Scale (SIOSS). Differences between the two groups were examined using the chi-square (χ²) test. *p < 0.05, **p < 0.01, ***p < 0.001.

### 3.3 Descriptive statistics and correlation analyses

#### 3.3.1 Independent samples t-test

In order to examine differences in psychological variables across levels of perceived social support, participants were divided into two groups: a low social support group (n = 187) and a high social support group (n = 317) based on the sample mean of the MSPSS total score.

Independent samples t-tests revealed that participants in the low social support group reported significantly higher levels of depression, anxiety, and suicidal ideation compared with those in the high social support group (all p <.001) (**Table 2**). 

**Table 2:** Group differences in key study variables.

Variables	Low social support group (n=187)	High social support group (n=317)	t-value	df	*P-value*
Depression	13.61 ± 6.73	9.09 ± 6.29	7.64	502	<0.001***
Anxiety	11.18 ± 5.30	7.78 ± 5.40	6.933	502	<0.001***
Suicidal ideation	13.50 ± 3.83	10.38 ± 3.50	9.398	502	<0.001***

*p < 0.05, **p < 0.01, ***p < 0.001 (two-tailed tests); M, mean; SD, standard deviation.

#### 3.3.2 Correlation analyses

Depressive symptoms and anxiety symptoms were highly positively correlated (r = 0.866, p < 0.01). Depressive symptoms were significantly and positively correlated with suicidal ideation (r = 0.661, p < 0.01), as were anxiety symptoms (r = 0.638, p < 0.01). Total perceived social support and its three dimensions—family support, friend support, and support from significant others—were all significantly and negatively correlated with depressive symptoms, anxiety symptoms, and suicidal ideation (r = −0.367 to −0.477, all p < 0.01). The three dimensions of social support were also significantly and positively correlated with one another and with total social support scores (r = 0.698–0.920, all p < 0.01), indicating strong associations among the MSPSS dimensions. These zero-order correlations provided preliminary evidence for subsequent analyses of the moderating effects of social support (**Table 3**).

**Table 3:** Correlations among study variables.

Variable	1	2	3	4	5	6	7
Depression	1						
Anxiety	.866^**^	1					
Suicidal ideation	.661^**^	.638^**^	1				
Social support	-.462^**^	-.418^**^	-.477^**^	1			
Family support	-.367^**^	-.364^**^	-.393^**^	.891^**^	1		
Support from friends	-.436^**^	-.371^**^	-.446^**^	.917^**^	.698^**^	1	
Support from significant others	-.438^**^	-.395^**^	-.470^**^	.920^**^	.754^**^	.809^**^	1

*p < 0.05, **p < 0.01, ***p < 0.001 (two-tailed tests); M, mean; SD, standard deviation.

### 3.4 Moderating effects of source-specific social support on the associations between depression, anxiety, and suicidal ideation

After adjusting for demographic covariates, hierarchical regression analyses were conducted. The VIF results for all models are presented in **Supplementary Table S3**. The VIFs for the main-effect variables were all below 5, whereas the highest VIF for the interaction terms was 6.4. These values did not indicate severe multicollinearity. The full-sample continuous interaction model simultaneously including depressive and anxiety symptoms was used as the primary model for evaluating the moderating effects of social support. The results of the full-sample hierarchical regression analyses are presented in **Table 4**.

**Table 4:** Primary moderation analysis.

Moderator	Model	Predictor	B (SE)	β	*p*	R²	ΔR²	ΔF
Perceived social support	Model 2	Depression	0.209 (0.039)	0.358	<0.001	0.5	0.48	157.309***
		Anxiety	0.173 (0.045)	0.251	<0.001			
		Perceived social support	−0.065 (0.011)	−0.208	<0.001			
	Model 3	Depression × Perceived social support	−0.133 (0.036)	−0.353	<0.001	0.522	0.022	11.479***
		Anxiety × Perceived social support	0.169 (0.035)	0.437	<0.001			
Family support	Model 2	Depression	0.234 (0.039)	0.401	<0.001	0.486	0.467	148.896***
		Anxiety	0.168 (0.046)	0.242	<0.001			
		Family support	−0.125 (0.028)	−0.155	<0.001			
	Model 3	Depression × Family support	−0.229 (0.081)	−0.220	0.005	0.498	0.012	5.836**
		Anxiety × Family support	0.021 (0.006)	0.166	<0.001			
Friend support	Model 2	Depression	0.207 (0.039)	0.355	<0.001	0.494	0.474	153.203***
		Anxiety	0.188 (0.046)	0.271	<0.001			
		Friend support	−0.165 (0.032)	−0.186	<0.001			
	Model 3	Depression × Friend support	−0.282 (0.095)	−0.256	=0.003	0.507	0.014	6.702**
		Anxiety × Friend support	0.027 (0.007)	0.193	<0.001			
Significant-other support	Model 2	Depression	0.207 (0.038)	0.356	<0.001	0.502	0.482	159.026***
		Anxiety	0.177 (0.045)	0.256	<0.001			
		Significant-other support	−0.182 (0.031)	−0.211	<0.001			
	Model 3	Depression × Significant-other support	−0.020 (0.009)	−0.181	0.027	0.512	0.011	5.329**
		Anxiety × Significant-other support	0.033 (0.011)	0.239	0.003			

All models were adjusted for seven demographic covariates. Continuous predictors were mean-centered before constructing interaction terms. In Model 3, depressive and anxiety symptoms were entered simultaneously with their respective interaction terms. **Table 4** reports the interaction effects from the joint moderation models. Conditional effects were examined separately using PROCESS Model 1 and are reported in **Table 5**. B, unstandardized regression coefficient; SE, standard error; β, standardized regression coefficient. *p < 0.05, **p < 0.01, ***p < 0.001.

Correlation analyses showed that total perceived social support and each of its three dimensions were significantly and negatively associated with depressive symptoms, anxiety symptoms, and suicidal ideation. Both depressive and anxiety symptoms were significantly and positively associated with suicidal ideation. The interaction terms between depressive symptoms and family support, friend support, and support from significant others were all significantly negative, indicating conventional buffering effects of the three sources of social support on the depression–suicidal ideation association. In contrast, the interaction terms between anxiety symptoms and each of the three sources of social support were all significantly positive, indicating reverse buffering effects and an asymmetric pattern across the two emotional associations.

For statistically significant interaction terms, simple slope analyses were conducted by estimating the conditional effects and their 95% confidence intervals (CIs) at the mean and ±1 SD of the moderator. Differences in the statistical significance of simple slopes across levels of the moderator were not considered sufficient, in themselves, to establish a moderating effect. Rather, the presence of moderation was determined strictly on the basis of the regression coefficient and confidence interval of the interaction term.

#### 3.4.1 Moderating effects in the full sample

After adjusting for demographic covariates, hierarchical regression analyses were conducted to examine whether perceived social support moderated the associations of depressive and anxiety symptoms with suicidal ideation. In the primary moderation analyses, depressive and anxiety symptoms were entered simultaneously in the same regression model. Separate hierarchical regression models were estimated for total perceived social support and for support from family, friends, and significant others. Continuous predictors were mean-centered before the interaction terms were calculated. The results are presented in **Table 4**.

Main effects. In the model including total perceived social support, depressive symptoms (β = 0.358, p < 0.001) and anxiety symptoms (β = 0.251, p < 0.001) were significantly and positively associated with suicidal ideation, whereas perceived social support was significantly and negatively associated with suicidal ideation (β = −0.208, p < 0.001). Similar patterns were observed in the models examining the three sources of social support separately. Depressive and anxiety symptoms were positively associated with suicidal ideation in the family-support model (β = 0.401 and 0.242, respectively; both p < 0.001), the friend-support model (β = 0.355 and 0.271, respectively; both p < 0.001), and the significant-other-support model (β = 0.356 and 0.256, respectively; both p < 0.001). In contrast, family support (β = −0.155), friend support (β = −0.186), and significant-other support (β = −0.211) were all significantly and negatively associated with suicidal ideation (all p < 0.001).

Interaction effects. In the primary joint moderation models, the interaction terms between depressive symptoms and perceived social support were all significantly negative. Specifically, significant interactions were observed for total perceived social support (β = −0.353, p < 0.001), family support (β = −0.220, p = 0.005), friend support (β = −0.256, p =0.003), and significant-other support (β = −0.181, p = 0.027). These findings indicate a conventional buffering pattern, whereby higher perceived social support was associated with a weaker positive association between depressive symptoms and suicidal ideation.

In contrast, the interaction terms between anxiety symptoms and perceived social support were all significantly positive. Significant interactions were observed for total perceived social support (β = 0.437, p < 0.001), family support (β = 0.166, p < 0.001), friend support (β = 0.193, p < 0.001), and significant-other support (β = 0.239, p = 0.003). These findings indicate a reverse-buffering pattern, whereby higher perceived social support was associated with a stronger positive association between anxiety symptoms and suicidal ideation.

The estimated interaction coefficients varied across the different social support measures. However, because formal statistical comparisons of the interaction coefficients were not conducted, these numerical differences should not be interpreted as evidence that one source of social support had a statistically stronger moderating effect than another.

Overall, the primary joint moderation analyses indicated an asymmetric moderation pattern: perceived social support showed a conventional buffering pattern in the association between depressive symptoms and suicidal ideation, but a reverse-buffering pattern in the association between anxiety symptoms and suicidal ideation.

To further characterize these interaction patterns, separate PROCESS Model 1 analyses were conducted for depressive symptoms and anxiety symptoms for each source of social support. These analyses estimated the conditional effects of depressive or anxiety symptoms on suicidal ideation at specified levels of social support and are reported in **Table 5**. Because these PROCESS analyses used separate model specifications in which depression or anxiety was entered as the focal predictor, their conditional-effect estimates should not be interpreted as coefficients from the primary joint moderation models reported in **Table 4**.

**Table 5:** Conditional effects of depression and anxiety on suicidal ideation across levels of social support.

Moderator	Predictor	Social support level	B	SE	t	p	95% CI
Family support	Depression	Low (−1 SD)	0.3047	0.0500	6.0892	<0.001	[0.2064, 0.4030]
		Mean	0.2409	0.0384	6.2742	<0.001	[0.1655, 0.3164]
		High (+1 SD)	0.1772	0.0499	3.5531	<0.001	[0.0792, 0.2751]
Family support	Anxiety	Low (−1 SD)	0.0740	0.0547	1.3545	0.1762	[−0.0334, 0.1815]
		Mean	0.1768	0.0464	3.8138	<0.001	[0.0857, 0.2679]
		High (+1 SD)	0.2795	0.0563	4.9609	<0.001	[0.1688, 0.3902]
Friend support	Depression	Low (−1 SD)	0.3209	0.0595	5.3903	<0.001	[0.2040, 0.4379]
		Mean	0.2311	0.0395	5.8565	<0.001	[0.1535, 0.3086]
		High (observed maximum)	0.1483	0.0488	3.0386	0.0025	[0.0524, 0.2443]
Friend support	Anxiety	Low (−1 SD)	0.0496	0.0591	0.8396	0.4015	[−0.0665, 0.1657]
		Mean	0.1722	0.0455	3.7859	<0.001	[0.0828, 0.2615]
		High (observed maximum)	0.2851	0.0525	5.4301	<0.001	[0.1820, 0.3883]
Significant-other support	Depression	Low (−1 SD)	0.3163	0.058	5.4561	<0.001	[0.2024, 0.4302]
		Mean	0.2267	0.0386	5.8728	<0.001	[0.1509, 0.3026]
		High (+1 SD)	0.1372	0.0517	2.6544	0.0082	[0.0356, 0.2387]
Significant-other support	Anxiety	Low (−1 SD)	−0.0029	0.0732	−0.0391	0.9688	[−0.1467, 0.1410]
		Mean	0.1498	0.0457	3.2757	0.0011	[0.0600, 0.2397]
		High (+1 SD)	0.3025	0.0609	4.9647	<0.001	[0.1828, 0.4222]

All models were adjusted for demographic covariates. B, unstandardized regression coefficient; CI, confidence interval. Conditional effects indicate the effects of depression or anxiety on suicidal ideation at different levels of social support. Low (−1 SD), mean, and high (+1 SD) represent the corresponding moderator levels. Because +1 SD for friend support exceeded the observed range, the observed maximum was used as the high level. Conditional effects were estimated using separate PROCESS Model 1 analyses for depression and anxiet.*p < 0.05, **p < 0.01, ***p < 0.001.

#### 3.4.2 Conditional effect analyses

To further characterize the significant interaction effects identified in the full-sample moderation models, conditional effects of depression and anxiety on suicidal ideation were examined at low (−1 SD), mean, and high (+1 SD) levels of each social support dimension. The results are presented in **Table 5**.

For the depression pathway, depression was positively associated with suicidal ideation across all levels of family support (B = 0.1772–0.3047, all p < 0.001), friend support (B = 0.1483–0.3209, all p ≤ 0.0025), and significant-other support (B = 0.1372–0.3163, all p ≤ 0.0082). Thus, the conditional effect of depression on suicidal ideation decreased as social support increased, while remaining statistically significant across the examined levels of support.

For the anxiety pathway, the conditional effect of anxiety on suicidal ideation increased with higher levels of all three sources of social support. For family support, the conditional effect was nonsignificant at the low level (B = 0.0740, p = 0.1762), but significant at the mean (B = 0.1768, p < 0.001) and high levels (B = 0.2795, p < 0.001). Similar patterns were observed for friend support, with conditional effects of B = 0.0496, 0.1722, and 0.2851 at low, mean, and high levels, respectively, and for significant-other support, with conditional effects of B = −0.0029, 0.1498, and 0.3025, respectively. For all three sources of support, the conditional effects were nonsignificant at the low level but became significant at the mean and high levels.

Overall, these conditional-effect analyses further characterized the asymmetric moderation pattern observed in the full-sample models: higher social support was associated with a weaker depression–suicidal ideation association but a stronger anxiety–suicidal ideation association. The conditional effects were interpreted together with the corresponding interaction terms rather than on the basis of differences in statistical significance between individual conditional effects.

#### 3.4.3 Exploratory subgroup analyses by perceived social support level

##### 3.4.3.1 Low social support group

To further examine the moderating effects across different levels of perceived social support, exploratory subgroup sensitivity analyses were conducted by stratifying participants into low- and high-social-support groups and performing separate regression analyses. In the low-social-support group, the interaction between family support and anxiety symptoms was significant and positive (β = 0.348, p = 0.001), as was the interaction between significant-other support and anxiety symptoms (β = 0.206, p = 0.037). In contrast, the interaction between friend support and anxiety symptoms was not statistically significant. Thus, significant interaction terms were observed for family support and significant-other support, whereas no significant interaction was observed for friend support. These findings suggest that the moderating effects of support sources showed different patterns within the low-social-support subgroup. However, the difference between statistically significant and nonsignificant interaction terms should not be interpreted as evidence that the corresponding moderating effects differed significantly in magnitude (**Table 6**). 

**Table 6:** Hierarchical regression analyses in the low social support group (N = 187).

Moderating variables	Variables	Standardizedβ	*P-value*
Family support	Anxiety	0.204	0.116
	Depression	0.597	<.001
	Family support	-0.288	<.001
	Anxiety × Family support	0.348	0.001
	Depression × Family support	0.047	0.714
Friends’ support	Anxiety	0.097	0.428
	Depression	0.57	<.001
	Friend support	-0.24	0.001
	Anxiety × Friend support	0.172	0.079
	Depression × Friend support	-0.206	0.125
Support from significant others	Anxiety	0.143	0.248
	Depression	0.515	<.001
	Significant other support	-0.331	<.001
Moderating variables	Anxiety × Significant other support	0.206	0.037
	Depression × Significant other support	0.161	0.12

The dependent variable was suicidal ideation score. All models were adjusted for demographic covariates, and all variables were mean-centered. β = standardized regression coefficient. *p < 0.05, **p < 0.01, ***p < 0.001.

##### 3.4.3.2 High social support group

In the high-social-support group, only the interaction between support from significant others and depressive symptoms was statistically significant and positive (β = 0.192, p = 0.040). The interaction between support from significant others and anxiety symptoms showed a trend toward significance (β = 0.153, p = 0.090), but did not reach the conventional significance threshold. The remaining interaction terms between the three sources of social support and depressive or anxiety symptoms were not statistically significant. These findings indicate variation in the observed interaction patterns within the high-social-support subgroup. Notably, the positive interaction between support from significant others and depressive symptoms was observed only in this exploratory subgroup analysis and was not consistent with the negative interaction observed for the same variables in the primary full-sample model. Therefore, this subgroup finding was interpreted cautiously and was not used to override the primary full-sample interaction result or to infer a statistically significant difference between support sources (**Table 7**). 

**Table 7:** Hierarchical regression analyses in the high social support group (N = 317).

Moderating variables	Variables	Standardizedβ	*P-value*
Family support	Anxiety	0.479	<.001
	Depression	0.24	0.004
	Family support	0.077	0.092
	Anxiety × Family support	-0.037	0.579
	Depression × Family support	-0.036	0.606
Friends’ support	Anxiety	0.401	<.001
	Depression	0.261	0.002
	Friend support	0.041	0.367
	Anxiety × Friend support	0.037	0.632
	Depression × Friend support	0.107	0.104
Support from significant others	Anxiety	0.313	0.005
	Depression	0.256	0.002
	Significant other support	0.095	0.037
Moderating variables	Anxiety × Significant other support	0.153	0.09
	Depression × Significant other support	0.192	0.04

The dependent variable was suicidal ideation score. All models were adjusted for demographic covariates, and all variables were mean-centered. β = standardized regression coefficient. *p < 0.05, **p < 0.01, ***p < 0.001.

#### 3.4.4 PHQ-8 robustness analysis

To address the potential bias arising from conceptual overlap between item 9 of the PHQ-9 and the suicidal ideation outcome, the primary moderation models were re-estimated in the full sample using the PHQ-8 in place of the PHQ-9. The complete model results are presented in **Supplementary Table S2**. The interaction terms between PHQ-8 depressive symptoms and family support, friend support, and support from significant others were all significantly negative, whereas the interaction terms between anxiety symptoms and the three sources of social support were all significantly positive. The direction and statistical significance of the interaction terms were consistent with those observed in the primary PHQ-9 models, indicating that the asymmetric moderating pattern remained robust after addressing the potential item-overlap concern.

#### 3.4.5 Single-predictor sensitivity analyses

Depressive and anxiety symptoms were highly correlated at the zero-order level (r = 0.866). To examine whether the observed moderating effects were sensitive to the inclusion of both symptom dimensions in the same model, two separate moderation models were constructed: one including depressive symptoms only and the other including anxiety symptoms only. Complete results are presented in **Supplementary Table S2-2**.

When depressive symptoms were included as the sole emotional predictor, none of the interaction terms between depressive symptoms and the three sources of social support reached statistical significance. Similarly, when anxiety symptoms were included as the sole emotional predictor, none of the interaction terms between anxiety symptoms and the three sources of social support reached statistical significance. The interaction terms involving family support (p = .0869) and significant-other support (p = .0913) approached but did not reach the conventional significance threshold of p < 0.05.

These findings indicate that the interaction effects observed in the primary joint models were not consistently reproduced when depressive and anxiety symptoms were examined separately. Given the high correlation between depressive and anxiety symptoms, the differences between the primary and single-predictor models suggest that the estimated moderation effects may be sensitive to model specification and the shared variance between depressive and anxiety symptoms. Therefore, the moderation findings should be interpreted with caution and in the context of the specific model specification.

#### 3.4.6 Extreme-group sensitivity analysis

To further assess the robustness of the findings, an extreme-group approach based on the upper and lower 27th percentiles of total social support was applied, and the hierarchical regression analyses were repeated. Because this approach excludes the middle 46% of participants, it substantially reduces the available sample size and statistical power. Therefore, these analyses were treated as secondary sensitivity explorations and were not used as the primary basis for inference. The results are presented in **Table 8**.

**Table 8:** Robustness analyses comparing mean-split and extreme-group approaches.

Source of support	Group	Mean-split β (p)	Extreme-group β (p)	Change in statistical significance	Observed pattern
Family support	Low	Anxiety × Family: 0.348 (.001)	Anxiety × Family: 0.046 (.830)	Significant → Not significant	Attenuated interaction
Family support	High	Anxiety × Family: −0.037 (.579)	Anxiety × Family: 0.441 (.013)	Not significant → Significant	Reverse buffering
Friend support	High	Depression × Friend: 0.107 (.104)	Depression × Friend: −0.499 (.006)	Not significant → Significant (negative)	Conventional buffering
Friend support	High	Anxiety × Friend: 0.037 (.632)	Anxiety × Friend: 0.338 (.026)	Not significant → Significant (positive)	Reverse buffering
Significant-other support	Low	Anxiety × Significant Other: 0.206 (.037)	Anxiety × Significant Other: 0.135 (.830)	Significant → Not significant	Attenuated interaction
Significant-other support	High	Depression × Significant Other: 0.192 (.040)	Depression × Significant Other: −0.116 (.715)	Significant → Not significant	Unstable effect
Significant-other support	High	Anxiety × Significant Other: 0.153 (.090)	Anxiety × Significant Other: 0.514 (.100)	Positive trend strengthened	Marginal reverse buffering

The mean-split approach divided participants into low- and high-social-support groups based on the sample mean of the total MSPSS score. The extreme-group approach classified participants into low-, medium-, and high-social-support groups based on the lower and upper 27th percentiles. β, standardized regression coefficient; p, significance level. *p < 0.05, **p < 0.01, ***p < 0.001.

Compared with the mean-split approach, the statistical significance of some interaction terms changed under the extreme-group classification. Specifically, the previously significant anxiety × family support interaction in the low-social-support group no longer reached statistical significance. In the high-social-support group, the interactions between friend support and both depressive symptoms and anxiety symptoms reached statistical significance. Overall, the specific significant interaction patterns varied across grouping strategies. These findings indicate variability in the observed interaction patterns across support sources and emotional symptom dimensions within the subgroup analyses; however, they should not be interpreted as evidence of statistically significant differences in moderating effects between support sources.

Taken together, the observed fluctuations in the significance of individual interaction terms across extreme-group classifications indicate that subgroup-level moderation effects were not fully stable. Accordingly, the primary conclusions of the study were based on the full-sample continuous interaction models rather than on the subgroup analyses.

### 3.5 Summary of hierarchical regression findings

Taken together, the primary full-sample continuous moderation analyses identified a consistent asymmetric interaction pattern across the three sources of perceived social support. The subgroup and sensitivity analyses provided complementary evidence regarding the robustness, model dependence, and potential heterogeneity of these effects. Across all three sources of perceived social support, interactions with depressive symptoms were negative, consistent with conventional buffering of the depression–suicidal ideation association, whereas interactions with anxiety symptoms were positive, consistent with reverse buffering of the anxiety–suicidal ideation association. This opposite-direction pattern across emotional pathways represents the central finding of the present study and provides an asymmetric extension of the conventional social support buffering framework. The interaction coefficients also varied across family, friend, and significant-other support, indicating potential source-specific heterogeneity in the magnitude of the observed moderation effects. These coefficient differences are described descriptively, as formal pairwise comparisons of interaction coefficients were not conducted.

The PHQ-8 substitution analysis reproduced the direction and statistical significance of the interaction terms observed in the primary PHQ-9 models, providing additional support for the robustness of the asymmetric pattern. In contrast, the single-predictor sensitivity analyses showed attenuation of the interaction effects when depressive and anxiety symptoms were modeled separately. Given their high correlation, this finding suggests that the estimated moderation effects may be sensitive to model specification and the substantial shared variance between the two emotional symptom dimensions. The mean-split and 27% extreme-group analyses further revealed variability in individual interaction terms across levels of perceived social support, providing exploratory evidence regarding the potential boundaries and heterogeneity of the observed moderation pattern.

Overall, the primary conclusions were derived from the full-sample continuous interaction models. The sensitivity and subgroup analyses did not replace the primary findings but provided complementary evidence regarding their robustness, model dependence, and potential variation across levels and sources of perceived social support.

## 4 Discussion

### 4.1 Summary of key findings

The present study examined the moderating role of source-specific perceived social support in the associations of depressive and anxiety symptoms with suicidal ideation among college students. In the primary full-sample continuous interaction models, which simultaneously included depressive and anxiety symptoms, both symptoms were positively associated with suicidal ideation, and an asymmetric moderation pattern of perceived social support was observed. However, sensitivity analyses showed that these interaction effects were sensitive to model specification, as none of the depression × support or anxiety × support interactions remained statistically significant when the corresponding symptom was modeled without the other. Given the high correlation between depressive and anxiety symptoms (r = .866) and the elevated interaction-term VIF values (up to 6.4), these moderation findings should therefore be interpreted cautiously as model-dependent associations rather than unequivocal and independent moderating effects.

Both depressive and anxiety symptoms were positively associated with suicidal ideation. In the depression–suicidal ideation association, the interaction terms for family support, friend support, and support from significant others were negative, consistent with a conventional buffering pattern. In contrast, the corresponding interaction terms in the anxiety–suicidal ideation association were positive, consistent with a reverse buffering pattern at the statistical interaction level. The interaction effects were examined separately for family, friend, and significant-other support, and the observed patterns varied across support sources. However, because no formal statistical comparisons between interaction coefficients were conducted, these differences should not be interpreted as evidence that the moderating effects differed significantly across support sources. These findings indicate that the moderating role of perceived social support may be associated with both the emotional symptom dimension and the source of support, although the statistical differences between support sources remain to be formally tested.

The primary interaction pattern remained consistent when PHQ-8 was used instead of PHQ-9, reducing concern that the findings were primarily attributable to the conceptual overlap between PHQ-9 item 9 and suicidal ideation. However, the interaction effects were attenuated when depressive and anxiety symptoms were modeled separately. Given their high correlation in the present sample (r = 0.866), this finding suggests that estimates of moderation may be sensitive to model specification and to the substantial shared variance between depressive and anxiety symptoms. The joint model therefore provides a more appropriate basis for interpreting the distinct associations of these two symptom dimensions with suicidal ideation, while this result should not be interpreted as evidence of a causal or synergistic mechanism between depression and anxiety.

The subgroup analyses based on mean splitting and the 27% extreme-group approach produced some variation in the statistical significance of individual interaction terms. Because categorization of a continuous moderator reduces information and the extreme-group approach substantially reduces sample size (20), these analyses were treated as exploratory sensitivity analyses rather than confirmatory hypothesis tests. In addition, multiple interaction tests were conducted without adjustment for multiple comparisons; therefore, subgroup findings should be considered hypothesis-generating. These exploratory variations do not alter the asymmetric interaction pattern observed in the primary full-sample models.

Importantly, the positive interaction terms observed in the anxiety pathway should be interpreted as an asymmetric statistical interaction rather than evidence that social support causes or aggravates suicidal ideation. The MSPSS assesses perceived availability of social support and does not directly measure the amount, quality, intensity, intrusiveness, or appropriateness of support actually received (10). Thus, the present findings cannot establish that participants experienced excessive or harmful support. The cross-sectional design likewise precludes causal conclusions regarding whether increasing or modifying social support would change suicidal ideation.

### 4.2 Possible explanations for the asymmetric moderation patterns across support sources

The observed pattern may be considered in light of the social support paradox and support-matching perspectives (22). Under the present statistical definition, negative interaction coefficients indicate that higher perceived support was associated with a weaker positive association between emotional symptoms and suicidal ideation, whereas positive coefficients indicate the opposite pattern. This statistical distinction does not imply that higher social support is intrinsically harmful.

Source-specific differences may partly reflect differences in the interpersonal contexts represented by family members, friends, and significant others (10). Family support may represent relatively stable interpersonal resources, whereas support from friends and significant others may involve more immediate and variable interpersonal interactions. From a support-matching perspective, the potential relevance of support may depend on whether perceived support corresponds to an individual’s needs and circumstances. However, the present study did not directly assess support quality, responsiveness, timing, intrusiveness, or support–need congruence. Therefore, these mechanisms remain hypotheses rather than explanations.

Differences between depressive and anxiety symptoms may also contribute to the asymmetric pattern. Depressive symptoms are commonly characterized by negative self-evaluation and hopelessness, whereas anxiety symptoms involve heightened threat sensitivity and concerns regarding uncertainty or lack of control. Such differences may partly shape how perceived social support is associated with suicidal ideation. Nevertheless, because the present study did not directly measure these cognitive or interpersonal processes, the underlying mechanisms cannot be determined from the current findings.

Accordingly, the reverse buffering pattern should be understood as evidence of heterogeneity in the statistical association between anxiety symptoms, perceived social support, and suicidal ideation rather than evidence of harmful social support. Longitudinal studies incorporating direct measures of support quality, perceived responsiveness, and support–need congruence are needed to test these potential mechanisms.

### 4.3 Interpretation of differences across grouping strategies

The mean-split and 27% extreme-group analyses yielded some differences in the significance of individual interaction terms. This variation is likely to reflect, at least in part, the statistical consequences of categorizing a continuous moderator. Categorization results in information loss, while the extreme-group approach excludes the middle portion of the sample and reduces statistical power. These factors can increase sampling variability and contribute to unstable subgroup estimates.

The subgroup results may reflect either sampling variability or potential differences in the associations across levels of perceived social support, but the present data cannot distinguish such heterogeneity from statistical variation caused by subgrouping. This is particularly relevant given the high correlation between depressive and anxiety symptoms, which may increase uncertainty in smaller subgroup models.

Therefore, the full-sample continuous interaction models should remain the primary basis for inference, whereas subgroup analyses should be regarded as exploratory evidence concerning the potential boundaries of the observed associations. Future studies should retain social support as a continuous construct and use larger samples to evaluate whether the asymmetric pattern varies systematically across different levels and sources of perceived social support.

### 4.4 Theoretical contributions and practical implications

The findings extend the conventional social support buffering framework by suggesting that the moderating role of perceived social support may not be uniform across emotional symptom dimensions. The depression–suicidal ideation association showed a buffering-consistent pattern, whereas the anxiety–suicidal ideation association showed a reverse buffering pattern. The interaction effects were examined separately across family, friend, and significant-other support, allowing the observed patterns for each support source to be described. However, because formal comparisons between interaction coefficients were not conducted, the present findings do not establish statistically significant differences in moderating effects across support sources. These findings broaden the buffering framework by emphasizing the potential importance of both emotional pathways and support-source heterogeneity.

The results also have methodological implications for research on suicidal ideation. Because depressive and anxiety symptoms showed substantial shared variance, simultaneously modeling both symptom dimensions may provide a more informative assessment of their potentially distinct associations with suicidal ideation than single-predictor models. However, this observation concerns model specification and should not be interpreted as evidence of a causal interaction between depression and anxiety.

From a practical perspective, the findings suggest that university mental health assessment should avoid assuming that higher perceived social support is uniformly associated with more favorable outcomes. Assessment may consider students’ emotional symptom profiles, perceived sources of support, and subjective experiences of available support. For students with depressive symptoms, family, peer, and significant-other support may represent potentially relevant psychosocial resources. For students with anxiety symptoms, clinicians may additionally assess how existing support is perceived and whether it is experienced as responsive and consistent with the student’s needs. These implications do not suggest reducing social support for students with anxiety, nor do they establish the effectiveness of any specific intervention.

More broadly, social support should be considered as one component of comprehensive suicide risk assessment rather than as an isolated protective factor. Longitudinal and intervention studies are required to determine whether tailoring support to individual emotional and interpersonal needs has measurable effects on suicidal ideation.

### 4.5 Limitations and future directions

Several limitations should be considered. First, the cross-sectional design precludes conclusions about temporal ordering or causality among depressive symptoms, anxiety symptoms, perceived social support, and suicidal ideation. Reciprocal associations are possible, including the possibility that suicidal ideation may influence subsequent perceptions of social support. Longitudinal and multi-wave studies are therefore needed to clarify temporal relationships.

Second, the MSPSS measures perceived availability of social support rather than objectively received support. The present study therefore cannot determine whether the observed patterns reflect differences in the amount, quality, intensity, or appropriateness of support actually received. Future studies should distinguish perceived from received support and directly assess support quality, responsiveness, and support–need congruence. In addition, the “significant others” dimension of the MSPSS does not specifically denote romantic partners. Because partner-specific support was not separately assessed, the present findings should not be interpreted as evidence concerning romantic partner support.

Third, the online convenience sample was restricted to college students and included a relatively high proportion of female and senior participants, which may limit generalizability. All major variables were assessed using self-report measures, which may also introduce response and common-method biases. Future research should recruit larger and more diverse samples and incorporate multiple assessment methods where feasible.

Fourth, multiple interaction terms and subgroup comparisons were examined without multiplicity correction, and subgroup categorization reduced information and statistical power. The resulting subgroup findings should therefore be interpreted cautiously. Future studies should preregister specific interaction hypotheses, retain continuous moderators where possible, and apply appropriate multiplicity-control procedures for confirmatory analyses.

Future research should therefore prioritize longitudinal designs, larger and more diverse samples, and direct measurement of both perceived and received social support. Such work may clarify whether the asymmetric interaction patterns observed here are temporally stable, identify the interpersonal and psychological processes that may account for differences in the observed patterns across support sources, and determine whether these statistical patterns have implications for suicide risk assessment or intervention. It is important to note that the present study was designed as a cross-sectional, questionnaire-based investigation and did not include a clinical intervention or crisis-management component. Accordingly, no specific psychological intervention was provided as part of the study protocol for participants reporting suicidal ideation. Future studies should consider incorporating appropriate risk-screening and referral procedures, consistent with relevant ethical and institutional requirements, to facilitate timely professional assessment and support for participants who may be at elevated risk. Such procedures may further strengthen the ethical safeguards and practical implications of research on suicidal ideation among college students.

## 5 Conclusion

The present study identified a potentially asymmetric pattern in the moderating role of social support in the associations between depressive and anxiety symptoms and suicidal ideation. In the primary full-sample continuous models, social support attenuated the association between depressive symptoms and suicidal ideation, whereas its interaction with anxiety symptoms was positive. Simple slope analyses further illustrated these conditional associations across different levels of social support. However, the interaction effects were attenuated when depressive and anxiety symptoms were modeled separately, indicating that the observed pattern was sensitive to model specification. Therefore, these findings should be regarded as preliminary evidence of a potentially asymmetric moderation pattern rather than definitive evidence for an extension of the conventional social support buffering model. Further longitudinal and prospective research is needed to replicate these findings and clarify the underlying mechanisms.

## Data Availability

The datasets presented in this article are not readily available because they contain sensitive personal information of human participants, and sharing of the raw data is restricted by the institutional ethics committee to protect participant confidentiality. Requests to access the datasets should be directed to Yanli Shi, shiyanli222@163.com.
