# Supplementary material for: Asymmetric reverse buffering patterns of source-specific social support in the associations between depression, anxiety, and suicidal ideation among college students
Source: Front Psychiatry. 2026 Sep 30;17:1930307. doi: 10.3389/fpsyt.2026.1930307 (PMC13661922; doi:10.3389/fpsyt.2026.1930307)
Supplement: Supplementary file 1 [file DataSheet1.pdf]

Supplementary Table S1-1.Hierarchical Regression Results for the Full Sample With Depression and Anxiety Entered Simultaneously: Perceived Social Support and Family Support

Moderator	Model	Predictor	β	*p*	B (SE)	95% CI for B	R^2	ΔR^2	ΔF
Perceived social support	Model 1	—	—	—	—	—	0.020	0.020	1.412
	Model 2	Depression	0.358	<0.001	0.209 (0.039)	0.134, 0.284	0.500	0.480	157.309***
		Anxiety	0.251	<0.001	0.173 (0.045)	0.084, 0.263	—	—	—
		Perceived social support	−0.208	<0.001	−0.065 (0.011)	−0.087, −0.042	—	—	—
	Model 3 (Interactions)	Depression	—	—	0.221 (0.038)	0.146, 0.295	0.522	0.022	11.479***
		Anxiety	—	—	0.165 (0.045)	0.076, 0.253	—	—	—
		Perceived social support	—	—	−0.083 (0.020)	−0.122, −0.044	—	—	—
		Depression × perceived social support	−0.353	<0.001	−0.133 (0.036)	−0.205, −0.062	—	—	—
		Anxiety × perceived social support	0.437	<0.001	0.169 (0.035)	0.100, 0.238	—	—	—
Family support	Model 1	—	—	—	—	—	0.020	0.020	1.412
	Model 2	Depression	0.401	<0.001	0.234 (0.039)	0.159, 0.310	0.486	0.467	148.896***
		Anxiety	0.242	<0.001	0.168 (0.046)	0.077, 0.258	—	—	—
		Family support	−0.155	<0.001	−0.125 (0.028)	−0.180, −0.069	—	—	—
	Model 3 (Interactions)	Depression	—	—	0.233 (0.038)	0.157, 0.308	0.498	0.012	5.836**
		Anxiety	—	—	0.177 (0.046)	0.086, 0.268	—	—	—
		Family support	—	—	−0.025 (0.051)	−0.125, 0.074	—	—	—
		Depression × family support	−0.220	0.005	−0.229 (0.081)	−0.387, −0.070	—	—	—
		Anxiety × family support	0.166	<0.001	0.021 (0.006)	0.009, 0.033	—	—	—

Note. Models were adjusted for seven demographic covariates, including sex and age. All continuous predictor variables were mean-centered before the interaction terms were constructed. β = standardized regression coefficient; B = unstandardized regression coefficient; SE = standard error; CI = confidence

interval. Negative interaction coefficients indicate that the association between depression/anxiety and suicidal ideation decreases as the moderator increases, whereas positive interaction coefficients indicate that the association increases as the moderator increases.* ***p*** **< 0.05, **** ***p*** **< 0.01, ***** ***p*** **< 0.001.**

Supplementary Table S1-2.Hierarchical Regression Results for the Full Sample With Depression and Anxiety Entered Simultaneously: Friend Support and Significant-Other Support

Moderator	Model	Predictor	β	*p*	B (SE)	95% CI for B	R^2	ΔR^2	ΔF	df_1
Friend support	Model 1	—	—	—	—	—	0.020	0.020	1.418	7
	Model 2: Main effects	Depression	0.355	<0.001	0.207 (0.039)	0.130, 0.284	0.494	0.474	153.203***	3
		Anxiety	0.271	<0.001	0.188 (0.046)	0.098, 0.278	—	—	—	—
		Friend support	−0.186	<0.001	−0.165 (0.032)	−0.228, −0.102	—	—	—	—
	Model 3: Interactions	Depression	—	—	0.225 (0.039)	0.149, 0.302	0.507	0.014	6.702**	2
		Anxiety	—	—	0.173 (0.046)	0.084, 0.263	—	—	—	—
		Friend support	—	—	−0.045 (0.061)	−0.165, 0.074	—	—	—	—
		Depression × friend support	−0.256	=0.003	−0.282 (0.095)	−0.469, −0.095	—	—	—	—
		Anxiety × friend support	0.193	<0.001	0.027 (0.007)	0.013, 0.042	—	—	—	—
Significant-other support	Model 1	—	—	—	—	—	0.019	0.019	1.391	7
	Model 2: Main effects	Depression	0.356	<0.001	0.207 (0.038)	0.132, 0.283	0.502	0.482	159.026***	3
		Anxiety	0.256	<0.001	0.177 (0.045)	0.088, 0.266	—	—	—	—
		Significant-other support	−0.211	<0.001	−0.182 (0.031)	−0.242, −0.121	—	—	—	—
	Model 3: Interactions	Depression	—	—	0.227 (0.039)	0.150, 0.304	0.512	0.011	5.329**	2
		Anxiety	—	—	0.150 (0.046)	0.060, 0.240	—	—	—	—
		Significant-other support	—	—	−0.191 (0.033)	−0.256, −0.126	—	—	—	—
		Depression ×	−0.18	0.027	−0.02	−0.03	—	—	—	—

Moderator	Model	Predictor	β	*p*	B (SE)	95% CI for B	R²	ΔR²	ΔF	df1
		significant-other support	1		0 (0.009)	6, −0.003				
		Anxiety × significant-other support	0.239	0.003	0.033 (0.011)	0.012, 0.055				

Note. Models were adjusted for seven demographic covariates, including sex and age. All continuous predictor variables were mean-centered before the interaction terms were constructed. β = standardized regression coefficient; B = unstandardized regression coefficient; SE = standard error; CI = confidence interval. Negative interaction coefficients indicate that the association between depression/anxiety and suicidal ideation decreases as the moderator increases, whereas positive interaction coefficients indicate that the association increases as the moderator increases. ****p* **< 0.05, *****p* **< 0.01, ******p* **< 0.001.

Supplementary Table S2-1. Sensitivity Analysis Using PHQ-8 Depression Scores

**Moderator**	**Model**	**Predictor**	**B (SE)**	**β**	**t**	**p**	**95% CI for B**	**R²**	**ΔR²**	**ΔF**
Family support	Model 1	Demographic covariates	—	—	—	—	—	0.019	0.019	1.391
	Model 2	PHQ-8 depression	0.241 (0.043)	0.357	5.236	<0.001	0.157, 0.326	0.479	0.46	145.149***
		Anxiety	0.181 (0.047)	0.254	3.797	<0.001	0.090, 0.273			
		Family support	−0.137 (0.030)	−0.212	−5.272	<0.001	−0.196, −0.078			
	Model 3	PHQ-8 depression	0.235 (0.043)	—	—	—	0.151, 0.319	0.487	0.008	3.792*
		Anxiety	0.171 (0.047)	—	—	—	0.079, 0.263			
		Family support	−0.131 (0.030)	—	—	—	−0.190, −0.072			
		PHQ-8 depression × Family support	−0.016 (0.007)	−0.196	−2.379	0.018	−0.031, −0.001			
		Anxiety × Family support	0.023 (0.008)	0.244	3.025	0.003	0.006, 0.040			
Friend support	Model 1	Demographic covariates	—	—	—	—	—	0.019	0.019	1.398
	Model 2	PHQ-8 depression	0.231 (0.044)	0.361	5.221	<0.001	0.144, 0.317	0.488	0.469	150.319***
		Anxiety	0.178 (0.047)	0.262	3.712	<0.001	0.086, 0.270			
		Friend support	−0.185 (0.035)	−0.211	−5.862	<0.001	−0.255, −0.116			
	Mod	PHQ-8	0.221	—	—	—	0.135,	0.49	0.01	4.814**

Moderator	Model	Predictor	B (SE)	β	t	p	95% CI for B	R^2	ΔR^2	ΔF
	el 3	depression	(0.044)				0.307	8		
		Anxiety	0.163 (0.047)	—	—	—	0.070, 0.255			
		Friend support	−0.178 (0.035)	—	—	—	−0.247, −0.109			
		PHQ-8 depression × Friend support	−0.024 (0.010)	−0.215	−2.468	0.028	−0.043, −0.004			
		Anxiety × Friend support	0.034 (0.011)	0.242	3.011	0.002	0.012, 0.056			
Significant-other support	Model 1	Demographic covariates	—	—	—	—	—	0.019	0.019	1.391
	Model 2	PHQ-8 depression	0.228 (0.043)	0.353	5.283	<0.001	0.143, 0.313	0.496	0.477	155.402***
		Anxiety	0.171 (0.046)	0.243	3.696	<0.001	0.080, 0.262			
		Significant-other support	−0.201 (0.033)	−0.234	−6.079	<0.001	−0.266, −0.136			
	Model 3	PHQ-8 depression	0.219 (0.043)	—	—	—	0.134, 0.304	0.506	0.01	4.844**
		Anxiety	0.156 (0.046)	—	—	—	0.065, 0.247			
		Significant-other support	−0.192 (0.033)	—	—	—	−0.257, −0.127			
		PHQ-8 depression × Significant-other support	−0.022 (0.010)	−0.181	−2.231	0.026	−0.041, −0.003			
		Anxiety × Significant-other support	0.033 (0.011)	0.235	2.957	0.003	0.011, 0.054			

Note. B = unstandardized regression coefficient; SE = standard error; β = standardized regression coefficient; CI = confidence interval. Model 1 included the seven demographic covariates. Model 2 additionally included depressive symptoms, anxiety symptoms, and the corresponding social support variable. Model 3 further included the interaction terms between depressive symptoms and social support and between anxiety symptoms and social support. All continuous predictors were mean-centered before construction of the interaction terms. ΔR^2 represents the change in R^2 associated with the variables entered at each step. $p < 0.05$, $p < 0.01$, $p < 0.001$.

Table S2-2.Interaction Effects Across Joint and Single-Predictor Model Specifications

Analysis condition	Social support dimension	Interaction term	B	β	p
Primary joint model: PHQ-9 depression + anxiety	Family support	Depression × Family support	−0.229	−0.220	0.005
	Friend support	Depression × Friend support	−0.282	−0.256	=.003
	Significant-other support	Depression × Significant-other support	−0.020	−0.181	0.027
	Family support	Anxiety × Family support	0.021	0.166	<.001
	Friend support	Anxiety × Friend support	0.027	0.193	<.001
	Significant-other support	Anxiety × Significant-other support	0.033	0.239	0.003
Robust joint model: PHQ-8 + anxiety	Family support	PHQ-8 × Family support	−0.016	−0.196	0.018
	Friend support	PHQ-8 × Friend support	−0.024	−0.215	0.028
	Significant-other support	PHQ-8 × Significant-other support	−0.022	−0.181	0.026
	Family support	Anxiety × Family support	0.023	0.244	0.003
	Friend support	Anxiety × Friend support	0.034	0.242	0.002
	Significant-other support	Anxiety × Significant-other support	0.033	0.235	0.003
Sensitivity model: Depression only	Family support	Depression × Family support	0.0027	—	0.4377
	Friend support	Depression × Friend support	0.0034	—	0.4055
	Significant-other support	Depression × Significant-other support	0.0045	—	0.2466
Sensitivity model: PHQ-8 only	Family support	PHQ-8 × Family support	0.0043	—	0.3005
	Friend support	PHQ-8 × Friend support	0.003	—	0.5136
	Significant-other support	PHQ-8 × Significant-other support	0.0045	—	0.3103
Sensitivity model: Anxiety only	Family support	Anxiety × Family support	0.0081	—	0.0869
	Friend support	Anxiety × Friend support	0.0076	—	0.146
	Significant-other support	Anxiety × Significant-other support	0.0084	—	0.0913

Note. B represents the unstandardized regression coefficient, and β represents the standardized regression coefficient. The primary joint model simultaneously included PHQ-9 depression and anxiety, whereas the robust joint model simultaneously included PHQ-8 depression and anxiety. Sensitivity models included depression, PHQ-8, or anxiety separately. All models were adjusted for the seven demographic covariates. "—" indicates that the standardized coefficient was not available in the corresponding output.

Table S3.Variance Inflation Factors (VIFs) and Tolerance Values for the Regression Models

Variable	FamilySupport VIF	Tolerance	FriendSupport VIF	Tolerance	Significant-OtherSupport VIF	Tolerance
Main effects						
Sex	1.148	0.871	1.155	0.866	1.141	0.876
Age	1.917	0.522	1.919	0.521	1.92	0.521
Only child	1.334	0.75	1.329	0.752	1.349	0.741
Place of origin	1.337	0.748	1.338	0.747	1.35	0.741
Monthly	1.272	0.786	1.287	0.777	1.279	0.782

Variable	FamilySupport VIF	Tolerance	FriendSupport VIF	Tolerance	Significant-OtherSupport VIF	Tolerance
living expenses						
Year of study	1.767	0.566	1.771	0.565	1.772	0.564
Major type	1.079	0.927	1.091	0.916	1.086	0.921
Centered depression	4.322	0.231	4.423	0.226	4.516	0.221
Centered anxiety	4.247	0.235	4.234	0.236	4.272	0.234
Centered social support	3.882	0.258	1.93	0.518	1.502	0.666
Interaction terms						
Depression ×Social Support	5.976	0.167	3.59	0.279	6.431	0.155
Anxiety ×Social Support	2.453	0.408	2.391	0.418	6.093	0.164

Note. VIF, variance inflation factor. Tolerance was calculated as 1/VIF. VIF and tolerance values were reported separately for the main effects and interaction terms. Values were used to assess potential multicollinearity among the predictors included in the moderation models.
